# Mr. Denmark's Case of Gastritis

**Published:** 1806-12

**Authors:** Alexander Denmark


### *To the Editors of the Medical and Physical Journal.*

Gentlemen,

Without eulogium upon the excellency of your publication, and the variety of information it embraces, I beg leave to communicate a Case of Gastritis, which came under

---

Philosophical Transactions.

under my management last cruize, and terminated fatally. We all know how much the Science of Medicine has to lament the secrecy observed by practitioners in general, in unsuccessful cases: a fact, in my opinion, possessing greater proneness to the obstruction of truth, and advancement of knowledge, than all the *chimeras* of the day,—than all the multiplicity of *flimsy fabrications,* hatched and produced more for the vain-glorious purpose of self-gratification, than for truth. I would not be understood to deprecate, or deemed desirous of damping that laudable emulation, which prompts us towards perfection, as far as aspiring and finite man possesses the power of attaining, and to the accomplishment of which, your Journal is so well adapted.

William Usher, marine, ætat. 19, complained on the morning of the 4th of August, in lat. 60°. N. therm. 37°. Atmosphere, clouded and foggy.—Was a florid and healthy young man. The most prominent symptoms were as follow:—Universal languor; sense of general debility; inaptitude to motion; wandering pains; stupor; loss of appetite; cold shiverings; with a very small and frequent pulse. Having reduced the temperature in a few days, from 60°, sailing to Greenland in quest of French frigates, attributed the febrile paroxysm to this cause, and gave an antimonial emetic, which operated almost instantaneously, and in the course of the day procured two evacuations per anum. Towards evening he appeared much worse.—Stupor increased; tottered when he attempted to walk; replied to interrogatories with hesitation, and seeming reluctance; so that both mental and muscular exertions appeared to be equally affected with the greatest torpor. Pains all over him as before, but especially in the chest on inspiration; and there was an anhelation like that perceived on immersing of a child in the cold bath; pulse 140 and contracted, but had something of a tightened or wirey feel; skin natural. V. S. ad ℥xij. upon which his pulse became slower, but remarkably feeble, succeeded by spasmodic twitchings in different parts. The anhelation gradually ceased, giving place to deep sighing; and syncope was nearly produced. His arm was now bound up, and he was placed in an horizontal posture. Emplast. Vesicat. sterno applicetur. I now discovered that he had worn a wet shirt the preceding day. 5th. Slept none all night. Keeps his eyes constantly closed; says it is painful to open them; has a dull diffused pain pain of the head; face much flushed; stupor and disinclination to speak; loaths both food and drink; pain of the chest diffused, but rather inclined to the right side; great prostration of strength and seeming anxiety. Pulse 160. Surface warmer than natural, and parched. V. S. ad ℥x. in bed, upon which the spasmodic symptoms recurred as before, and the pulse sunk. Aq. hordei vel infus. theæ. ad libit. P. M. had one stool and vomited in the course of the day; complains of pain on pressing the hypogastric region. I now felt confirmed in my preconception, that the disease was *Gastritis.*

Rep. V. S. ad ℥x. Pulse sunk as formerly. All the blood hitherto drawn, when cold, appeared one solid mass of crassamentum, adhering firmly to the sides of the cup.

6th. He points distinctly to the principal pain in the region of the ventriculus, and complains of burning heat at the præcordia, with pain on pressure; vomits more frequently, rejecting almost every thing taken; pain in the posterior part of his head. Enema è decoct. Lini injiciend. et repetat. bis in die. P. M. symptoms aggravated. Extreme anxiety with occasional sobbing, and a desire to sit up; says he feels "numb all over." Vomiting excited on every attempt to swallow; pulse 130; rep. V. S. ad ℥x. et emp. vesicat. præcord. applic. Pulse bore the bleeding well. Ordered a spoonful of chicken broth to be given occasionally through the night.

7th. The blood drawn last night exhibits the buff surface, but it does not adhere to the side of the cup; slept two or three hours in the night, and has rejected nothing taken since; pulse continues at 130; complains chiefly of a chorded feel in the abdomen. Enema and chicken broth as before. P. M. pulse 130, but smaller; feet rather cold; wishes much to get up, and cries in consequence of being refused: has vomited nothing to day; complains but little of pain:—An enema of broth, with three grains of opium: et cap. opii gr. ß. confect. aromat. gr. v. Emp. epispast. tibiis applicantur. Two hours after this, his pulse increased to 144: heat on the surface increased; respiration quickened; says he feels "quite brave;" retained only part of the enema; had one small evacuation after it; seems inclined to doze.

8th. Pulse 144 and feeble; felt little pain from the blisters on his legs. Rep. enema. Refuses to swallow the least thing offered. P. M. could not resist indulging him in his solicitations, to get out of bed for an hour; when he returned at five o'clock, he was greatly exhausted; pulse hardly hardly perceptible, and about 160; great stupor and disposition to coma; had no vomiting to day, but he swallowed nothing. At seven, rep. enema with two grs. of opium; at nine, pulse very small, and 160; in other respects the same; got him to swallow one quarter grain of opium, with some sago gruel, which he retained.

9th. Find him more susceptible in his mental feelings; talks with more animation; pulse 144; drank half a cup of tea; says he feels but little pain at his stomach; gave a sago enema. At eight P. M. pulse remarkably frequent, fluttering, and small; enema repeated with gr. ij. opii; retained only part of it; stupor increased; appears free from pain, but says he feels quite numb; at nine, gave him opii gr. one quarter, and some tea, which he retained; keeps his eyes shut. At twelve, pulse at the wrist scarcely distinguishable; respiration, short and moaning; gave him some warm wine; has had the rattle in his throat all the evening. A clammy sweat broke out over the face and neck; he gradually sunk away, and died at four o'clock on the morning of the 10th.

On dissection, the whole of the internal coat of the stomach evinced a previously most highly inflamed state; some parts of it streaked with a purplish hue, similar to what we see in the tulip-leaf, and approaching near to that in colour; it was lined with mucous, and quite empty. The intestines contained nothing but flatus; and they were inflated to an enormous extent.

The most prominent practical points to be collated in this Case are; — 1st. The inflammation having been occasioned by the application of cold to the surface. 2dly. Its appearing to be combined with Pneumonia during the first two days, though not indicated by the pulse.

3dly. The pulse bearing the three first evacuations so ill: which, I think, induced too early an use of opium. There was no hickup, nor any erysipelatous inflammation of the fauces. Would a further use of the lancet, during the first three days, the warm bath, and abstinence from opium till a later period, have answered better? Was not the pneumonic affection symptomatic?

I am, &c.

ALEXANDER DENMARK.

